# Mitochondria transfer enhances proliferation, migration, and osteogenic differentiation of bone marrow mesenchymal stem cell and promotes bone defect healing

**DOI:** 10.1186/s13287-020-01704-9

**Published:** 2020-06-25

**Authors:** Yusi Guo, Xiaopei Chi, Yifan Wang, Boon Chin Heng, Yan Wei, Xuehui Zhang, Han Zhao, Ying Yin, Xuliang Deng

**Affiliations:** 1 National Engineering Laboratory for Digital and Material Technology of Stomatology, NMPA Key Laboratory for Dental Materials & Beijing Laboratory of Biomedical Materials, Peking University School and Hospital of Stomatology, Beijing, 100081 People’s Republic of China; 2 Department of Prosthodontics, Peking University School and Hospital of Stomatology, Beijing, 10081 People’s Republic of China; 3 Department of Geriatric Dentistry, Peking University School and Hospital of Stomatology, Beijing, 100081 People’s Republic of China; 4 Central Laboratory, Peking University School and Hospital of Stomatology, Beijing, 100081 People’s Republic of China; 5 Department of Dental Materials & Dental Medical Devices Testing Centre, Peking University School and Hospital of Stomatology, Beijing, 100081 People’s Republic of China; 6 Xiangya Stomatological Hospital, Central South University, Changsha, 410078 People’s Republic of China

**Keywords:** Mitochondria, Mitochondria transfer, BMSC function, Proliferation, Stem cell migration, Osteogenic differentiation, Metabolism

## Abstract

**Background:**

Bone marrow-derived mesenchymal stem cell (BMSC) transplantation is considered a promising therapeutic approach for bone defect repair. However, during the transplantation procedure, the functions and viability of BMSCs may be impaired due to extended durations of in vitro culture, aging, and disease conditions of patients. Inspired by spontaneous intercellular mitochondria transfer that naturally occurs within injured tissues to rescue cellular or tissue function, we investigated whether artificial mitochondria transfer into pre-transplant BMSCs in vitro could improve cellular function and enhance their therapeutic effects on bone defect repair in situ.

**Methods:**

Mitochondria were isolated from donor BMSCs and transferred into recipient BMSCs of the same batch and passage. Subsequently, changes in proliferative capacity and cell senescence were evaluated by live cell imaging, Cell Counting Kit-8 assay, cell cycle analysis, Ki67 staining, qPCR and Western blot analysis of c-Myc expression, and β-galactosidase staining. Migration ability was evaluated by the transwell migration assay, wound scratch healing, and cell motility tests. Alkaline phosphatase (ALP) staining, Alizarin Red staining, and combined with qPCR and Western blot analyses of Runx2 and BMP2 were performed to elucidate the effects of mitochondria transfer on the osteogenic potential of BMSCs in vitro. After that, in vivo experiments were performed by transplanting mitochondria-recipient BMSCs into a rat cranial critical-size bone defect model. Micro CT scanning and histological analysis were conducted at 4 and 8 weeks after transplantation to evaluate osteogenesis in situ. Finally, in order to establish the correlation between cellular behavioral changes and aerobic metabolism, OXPHOS (oxidative phosphorylation) and ATP production were assessed and inhibition of aerobic respiration by oligomycin was performed.

**Results:**

Mitochondria-recipient BMSCs exhibited significantly enhanced proliferation and migration, and increased osteogenesis upon osteogenic induction. The in vivo results showed more new bone formation after transplantation of mitochondria-recipient BMSCs in situ. Increased OXPHOS activity and ATP production were observed, which upon inhibition by oligomycin attenuated the enhancement of proliferation, migration, and osteogenic differentiation induced by mitochondria transfer.

**Conclusions:**

Mitochondria transfer is a feasible technique to enhance BMSC function in vitro and promote bone defect repair in situ through the upregulation of aerobic metabolism. The results indicated that mitochondria transfer may be a novel promising technique for optimizing stem cell therapeutic function.

## Background

Mesenchymal stem cells (MSCs) are multipotent, self-renewing adult stem cells that can differentiate into a variety of tissues [1, 2]. MSCs are considered to be particularly promising seed cells for bone tissue engineering due to their ease of isolation from bone marrow (bone marrow-derived mesenchymal stem cells (BMSCs)) or adipose tissue and can readily be expanded in vitro to sufficient numbers for clinical applications [3, 4]. Nevertheless, the functional properties of BMSCs might be impaired after isolation and cultivation for extended durations in vitro [5], or due to aging or disease conditions of the donor patients [6]. Of particular concern are their (i) survivabilility after transplantation, (ii) proliferative capacity, and (iii) osteogenic differentiation potential. Thus, modifying BMSCs to enhance these functions has become a major focus of recent research on stem cell-mediated bone regeneration.

Various strategies have been attempted to enhance the functions of engrafted stem cells. For example, pre-conditioning cells with drugs such as Rapamycin [7], and cytokines like TGF-β1 [8] or TNF-α [9], were able to promote osteogenesis, as well as enhance mobilization and proliferation of MSCs. But there are intrinsic drawbacks and challenges to be overcome, such as determining the optimal dosages or potential side effects. Other studies utilized genetic engineering to enhance MSCs function [6]. For example, MSCs that were genetically engineered to overexpress BMP2 have been shown to promote bone regeneration in the rat and mouse model [10, 11], and MSCs transduced to overexpress CXCR4 were able to increase bone strength in a murine osteoporosis model [12], as well as prevent bone loss in ovariectomized mice [13]. Nevertheless, there are numerous technical challenges and safety concerns pertaining to utilizing genetically engineered MSCs in clinical therapy, particularly the difficulties faced in developing clinical grade vectors [14]. Hence, to date, there are still many drawbacks in most current strategies that have attempted to improve the functionality of BMSCs.

Many natural phenomena that spontaneously occur in the human body during healing have inspired novel theraputic strategies. It is well-known that when tissues or organs undergo stress or injury, intercellular mitochondria transfer spontaneously occurs to rescue their function. For example, astrocytes in mice have been observed to release functional mitochondria that enter neurons and contribute to endogenous neuroprotective and neurorecovery mechanisms after stroke [15]. Similarly, BMSCs have been documented to transfer mitochondria to alveolar epithelial cells to protect against endotoxin-induced [16] or cigarette-induced [17] lung injury. Inspired by such naturally occurring phenomena, we hypothesize that artificial mitochondria transfer in vitro might be able to improve BMSC functions and enhance the efficacy of BMSC-based bone regeneration.

Hence, in this study, we aimed to investigate whether autologous mitochondria transfer to BMSCs prior to transplantation could improve their cellular function and enhance their capacity to promote bone regeneration in situ. We isolated mitochondria from donor BMSCs and transferred these into recipient BMSCs at the same passage. Functional properties of recipient BMSCs including proliferation, migration, and osteogenic differentiation were then evaluated. Subsequently, an in vivo study was performed by implanting the mitochondria-recipient BMSCs into a rat critical-size cranial bone defect model to evaluate the effects of mitochondria transfer on BMSC-mediated bone defect repair. Finally, we investigated the possible relationship between metabolic and functional changes within mitochondria-recipient BMSCs following transplantation, to uncover the underlying mechanisms of the observed enhancement in bone regeneration efficacy.

## Methods

### Cell culture and mitochondria transfer

Sprague-Dawley rat BMSCs were purchased from Cyagen Biosciences (Guangzhou, China). Cells were cultured in α-MEM (Hyclone SH30265.01B) supplemented with 10% (v/v) fetal bovine serum (FBS) (Gibco 10099141) and 1% (v/v) penicillin-streptomycin solution (Gibco 15140122). Cells were cultured in a 37 °C, 5% CO_2_ incubator with a humidity level of 90–95%. The culture medium was refreshed every 1 to 2 days. All cells utilized in experiments were between passage 4–7 (except for the senescence-associated β-galactosidase staining assay). Cellular and mitochondrial exposure to EDTA were avoided at all steps in the experiments. For mitochondria transfer, both donor and recipient BMSCs were seeded into a 6-well plate at 2 × 10^5^ cells per well, with donor BMSCs being harvested after 36 h. The Mitochondria Isolation Kit for Cultured Cells (ThermoFisher, Rockford, Illinois, USA) was utilized to isolate mitochondria from donor BMSCs according to the manufacturer’s instruction. A series of differential centrifugation steps were carried out to separate the mitochondrial and cytosolic fractions. Isolated mitochondria were directly resuspended in 1 mL of complete medium and kept on ice before transfer. The supernatant of the recipient BMSCs was removed, and the mitochondria suspension was added slowly close to the bottom of the well. As for control BMSCs, the supernatant was also removed, and 1 mL of medium without mitochondria was added instead. The whole plate was centrifuged at 1500 rcf at 4 °C for 15 min, placed within a 37 °C incubator for 2 h, and centrifuged under the same conditions again, in order to facilitate cellular mitochondria uptake. The cells were then placed back into a 37 °C incubator for 24 h before subsequent experiments.

### Laser scanning confocal microscopy

For validation, MitoTracker® Deep Red FM (absorption/emission ~ 644/665 nm) was utilized at a concentration of 500 nM, to label mitochondria of donor MSCs before isolation. Samples were then fixed with 4% (w/v) paraformaldehyde for 15 min, prior to examination under a laser confocal microscope (Leica). EDTA-free trypsin was utilized in all experiments in order to prevent membrane damage and MitoTracker leakage.

### Flow cytometry

For quantitative validation, MitoTracker® Green FM (absorption/emission ~ 490/516 nm) was utilized at a concentration of 100 nM to label mitochondria in donor MSCs before isolation. Quantification of mitochondria was carried out by using a BD FACSAria™ III (Becton Dickinson, Franklin Lakes, NJ, USA) flow cytometer, with at least 10,000 events for each sample and analysis being carried out with the BD FACSDiva™ software. Readings (in duplicates) for mean fluorescence intensity (MFI) in the FITC emission region were recorded, and regression analysis was performed with GraphPad Prism 6.01.

### Proliferation curve and CCK8 assay

To construct the proliferation curve, control and recipient BMSCs were seeded at a density of 1 × 10^5^ cells per well of a 6-well plate, 24 h after mitochondria transfer. Then, the plate was placed into the Live Cell Imaging System, with images of each well being captured every 2 h. The cell confluency of each image was calculated with IncuCyte software. For the CCK8 proliferation assay, BMSCs (1 × 10^5^ cells per well) were seeded in 12-well plates and then incubated at 37 °C with 5% CO_2_. After 48 h, the medium was replaced with culture medium containing 10% (v/v) CCK8 kit (Dojindo, Shanghai China) solution, followed by incubation at 37 °C for an additional 2 h. The supernatant was then placed into a 96-well plate, and the absorbance was then measured using a microplate reader at 450 nm, with 3 replicates per group.

### Cell cycle analysis

Modulation of the cell cycle was analyzed at 24 h after mitochondria transfer. After trypsinization and rinsing with PBS, the cells were fixed in 70% (v/v) ethanol and incubated on ice for 15 min. Then, the cells were labeled with propidium iodide (PI)/RNase staining solution (#4087, Cell Signaling Technology, The Netherlands) and incubated at room temperature for 15 min. Cells were analyzed using BD FACSAria™ III (Becton Dickinson, NJ, USA). Data analysis was performed using FlowJo 7.6. Histograms were constructed with GraphPad Prism 6.01.

### Immunofluorescence analysis

Samples were rinsed with phosphate-buffered saline (PBS) and fixed in 4% (w/v) paraformaldehyde for 15 min. After fixation, we washed the samples three times with PBS for 5 min each time. Then, samples were permeabilized with 0.1% (w/v) Triton X-100 (diluted with PBS) for 10 min and blocked with 3% (w/v) bovine serum albumin (BSA; diluted with PBS) for 1 h to minimize non-specific staining. After the removal of the permeabilization solution, samples were rinsed and washed with PBS again. The above procedures were carried out at room temperature. Samples were then incubated with the primary antibody—Rabbit Anti-Ki67 antibody (1:250; ab16667; abcam) in 3% (w/v) BSA overnight at 4 °C. After thorough rinsing with PBS to remove excess antibodies, the cells were incubated with Goat Anti-Rabbit IgG H&L (Alexa Fluor® 488) pre-adsorbed secondary antibody (2 μg/mL; ab150081; abcam) for 1 h in darkness. 4′,6-Diamidino-2-phenylindole (DAPI; Sigma) was used to stain cellular nuclei. Images of three random fields of vision were captured with a confocal laser scanning microscope (Leica). Ki67-positive cells in each group (*n* = 3) were quantified with Image-Pro Plus, and GraphPad Prism 6.01 was used for statistical analysis.

### Senescence-associated β-galactosidase staining

Expression of senescence-associated β-galactosidase (SA-b-gal) activity was evaluated in different passages of BMSC using the SA-b-gal staining kit (Beyotime, Shanghai, China). Recipient and control BMSCs at passage 6 to 9 were seeded in a 6-well plate at 20 × 10^5^ cells per well. When cells reached 90% confluence, the medium was discarded, and the cells were rinsed with PBS once, prior to fixing with 4% (w/v) paraformaldehyde for 15 min, and subsequent rinsing with PBS for a further three times. Then 1 mL of working solution was added to the plate, which was maintained at 37 °C overnight away from light. The senescent cells in each group (*n* = 3) were observed under an optical microscope and images from three random fields of vision were captured. The Image-Pro Plus software was used for cell counting, and GraphPad Prism 6.01 was used for statistical analysis.

### CyQUANT™ cell proliferation assay

BMSCs were seeded onto a 24 well glass-bottom plate with 4 × 10^4^ cells per well in 3 replicates. The cells were incubated for 4 h to allow adhesion prior to staining with the CyQUANT® NF Cell Proliferation Assay Kit (Invitrogen, USA) for another 30 min. We quantified the positively stained cells within each group (*n* = 3) from three random fields of vision under fluorescence microscopy. The Image-Pro Plus software was used for cell counting, while GraphPad Prism 6.01 was used for statistical analysis.

### Vertical migration test, scratch wound healing, and cell tracking

Vertical migration assays were performed in 6.5 mm Transwell® with 8.0 μm Pore Polycarbonate Membrane Inserts (Corning, NY, USA). About 8000 cells (suspended in 200 μL/well) were seeded into the upper chambers in α-MEM without FBS, with the lower chamber containing 600 μL of complete α-MEM (10% v/v FBS). After 6 h, cells that have migrated to the bottom layer were washed and fixed with 4% (w/v) paraformaldehyde for 15 min, while cells remaining in the upper chamber were removed. The chambers were then immersed into 0.05% (w/v) crystal violet dyes to stain cells at the bottom. Five micrographs were taken for each chamber and the cell number (3 replicate readings per group) were counted manually and statistical analysis as then performed using the GraphPad Prism 6.01 software. Both scratch wound healing and cell tracking assays were carried out with the Cell IQ live cell kinetic imaging & quantification system (CM technologies, Colorado, USA). For the scratch wound-healing assay, cells were seeded at a density of 2 × 10^5^ cells/well (in 24-well plates), and a scratch was made on the cell monolayer 12 h later. After being washed three times with serum-free medium, the cells were placed into the Cell IQ system and observed for another 24 h. For cell tracking, the cells were seeded at 3000 cells/well (in 24-well plates) for 5 h, prior to being transferred into the Cell IQ system. All wells were imaged every 10 min. The images were analyzed using a Cell IQ Analyzer. To avoid the effects of proliferation, serum-free culture medium was used in the scratch wound healing and cell tracking assays. Cell migration was expressed as follows: new scratch width/initial scratch width × 100%.

### Alkaline phosphatase (ALP) and Alizarin Red S staining

Osteogenic induction was carried out by culturing cells in osteogenic differentiation medium (Cyagen Biosciences) containing 10% (v/v) FBS, 1% (v/v) penicillin-streptomycin, 2 mM l-glutamine, 50 μM ascorbate, 10 mM β-glycerophosphate, and 100 nM dexamethasone. The culture medium was changed every 2–3 days. The BMSCs were induced in the osteogenic differentiation medium for 4, 7, and 14 days. The cells were then washed twice in PBS, fixed with 4% (w/v) paraformaldehyde for 15 min, and then stained with alkaline phosphatase (ALP) staining solution (A059-2-2, Nanjing Jiancheng Bioengineering Institute, Nanjing, China), after 4 and 7 days of induction, according to the manufacturer’s instructions. Measurement of ALP activity was performed with an Alkaline Phosphatase Assay Kit (Beyotime, Shanghai, China) following the manufacturer’s instructions (*n* = 3). Alizarin Red S staining was carried out after 14 days of induction. After fixing with ice-cold 70% (v/v) ethanol, each well was treated with 1 mL of freshly prepared 3% (w/v) Alizarin Red S solution (Sigma-Aldrich, Missouri, USA) and incubated in the dark for 30 min. For quantitative analysis, three replicate absorbance readings for each group was measured at 595 nm following destaining with 10% (v/v) cetylpyridinium chloride monohydrate (Sigma-Aldrich) for 20 min.

### Real-time quantitative RT-PCR analysis

Total RNA extraction was carried out using TRIzol Reagent (Invitrogen, USA) according to the manufacturer’s instructions. Amplifications were then performed with the different primers. The quality and quantity of the RNA obtained were subjected to spectrophotometric analysis using a bio-photometer (Thermo Scientific™ NanoDrop8000). The RNA was then reversed-transcribed into complementary DNA (cDNA) using a Reverse Transcription kit (Takara Bio Inc., Japan). Quantitative real-time polymerase chain reaction (qPCR) was performed with the SYBR Green PCR reagent kit (Roche, Germany) on an ABI QuantStudio 3 Real-Time PCR System (Applied Biosystems, Foster City, CA, USA). The primer sequences are listed in Table 1. All values were normalized to GAPDH.

**Table 1:** Primer sequences used for quantitative real-time PCR analysis

Gene	Forward sequence (5′-3′)	Reward sequence (5′-3′)
GAPDH	GGGTCGGTGTGAACGGATTTGG	GCCGTGGGTAGAGTCATACTGGAAC
C-Myc	AACCCGACAGTCACGACGATG	GCTCTGCTGTTGCTGGTGATAG
Runx2	GAGATTTGTAGGCCGGAGCG	CCCTAAATCACTGAGGCGGT
BMP2	TGCTCAGCTTCCATCACGAAG	TCTGGAGCTCTGCAGATGTGA

### Western blot analysis

The cultured cells were lysed with RIPA lysis buffer (Beyotime, Shanghai, China) supplemented with protease inhibitor cocktail (ThermoFisher, Rockford, Illinois, USA) on ice. The protein concentration was quantified using a BCA protein assay kit (Beyotime). Six times SDS Sample Loading Buffer (P0015F; Beyotime) was added to the protein before heating at 100 °C for 5 min. Then, the total protein extract (30 μg) was separated by 10% (w/v) sodium dodecylsulfate polyacrylamide gel electrophoresis, and proteins were transferred to a PVDF membrane. The membranes were blocked by 5% (w/v) skimmed milk and incubated with the primary antibody at 4 °C overnight, followed by incubation with a secondary antibody conjugated with horseradish peroxidase (HRP). Autoradiography was performed with an eECL Western Blot Kit (CoWin Bio., Jiangsu, China) on a film exposure machine. The primary antibodies C-Myc (ab39688), Runx2 (ab23981), and BMP2 (ab14933) were purchased from Abcam. The primary antibody against β-Actin (AF0003) and secondary antibody HRP-labeled IgG (A0208, A0216) were purchased from Beyotime, China. β-Actin was utilized as the protein loading control. The protein expression levels were normalized to β-Actin.

### Cell aerobic metabolism measurements

#### Measurement of OXPHOS activity

Cells were trypsinized and seeded on a SeaHorse® 24-well XF-24 plate at a density of about 10,000 per well in XF base medium supplemented with 1 g/L glucose, 1 mM sodium pyruvate, and 2 mM glutamine and were then placed into a SeaHorse XF Extracellular 24 Flux Analyzer, in order to measure their oxygen consumption rate (OCR). Mitochondrial respiration inhibitors —1.0 μM oligomycin, 1.0 μM carbonyl cyanid-4 phenylhydrazone (FCCP), 0.5 μM antimycin A and rotenone—were used to treat the cells in the system, and OCR was measured before and after treatment with the inhibitors, for determination of basal respiration, ATP production, maximal respiration, and spare respiratory capacity. All results were normalized to the number of cells per well, counted immediately after detection.

#### Measurement of ATP production

Measurement of ATP production was performed on 10,000 cells per group using the ATPlite luminescent detection assay (Perkin Elmer), according to the manufacturer’s instructions. Measurements were expressed as relative luciferase units (RLU) and calculated as fold of RLU, as measured in the control group.

### Animal experiments

#### Animals and surgical procedures

Forty 7-week-old male Sprague-Dawley (SD) rats were used in this study. The experimental protocol was approved by the Animal Care and Use Committee of Peking University. To establish the cranial defect model, the dorsal cranium was surgically exposed after the rats were anesthetized by phenobarbital sodium (100 mg/kg) via intraperitoneal injections. Two critical-sized full thickness bone defects (5 mm in diameter) on each side of the parietal bone were performed by a saline-cooled trephine drill. There were four groups (*n* = 5): blank—without any implantation; NC (negative control), both sides filled with Matrigel only; control—both sides filled with Matrigel and 5 × 10^5^ control BMSCs for each defect; and treatment—both sides filled with Matrigel and 5 × 10^5^ BMSCs after mitochondria transfer.

### Micro-CT scanning evaluation

At 4 and 8 weeks post implantation, calvaria samples were harvested and fixed in 4% (w/v) paraformaldehyde for 24 h at room temperature. The specimens were then examined using Viva40 micro-CT scanner (Scanco Medical. AG®). Bone volume was analyzed, and 3D reconstruction was built based on the processed images using Scanco® software.

### Histological analysis

Following micro CT analysis, rat skulls were decalcified and paraffin-embedded. Histomorphology analysis was performed on 5-μm-thick histology sections of the central portion of the skull defect. The sections were then subjected to hematoxylin and eosin (H&E) and Masson’s trichrome staining, according to the manufacturer’s protocols. Images were captured using an Olympus D70 camera mounted on a Nikon Eclipse E800 microscope.

## Results

### Successful transfer of mitochondria into BMSCs in vitro

**Fig. 1:**
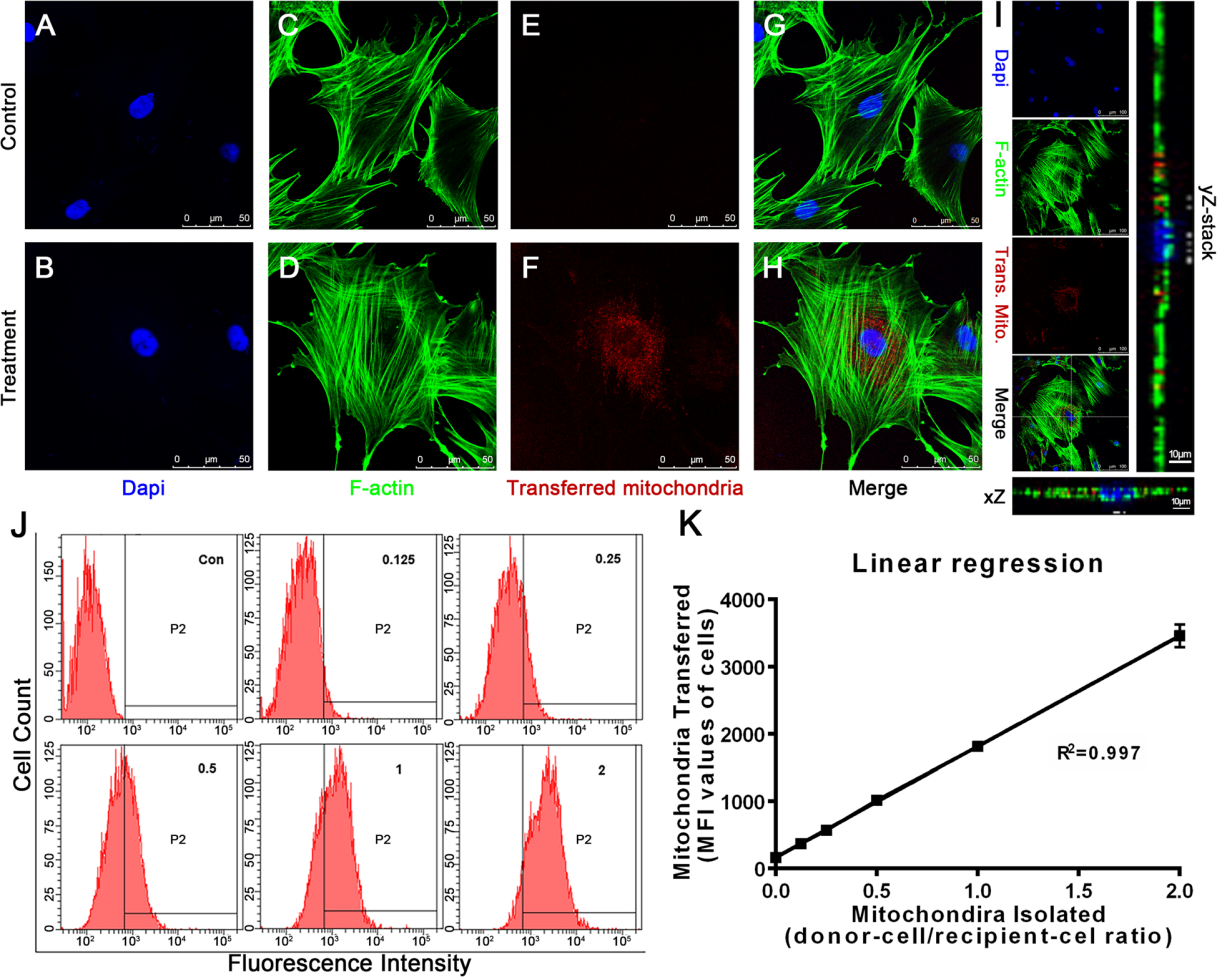
Uptake of donor mitochondria by recipient BMSCs increased in a dose-dependent manner. Confocal immunofluorescence staining of nuclei with DAPI in BMSCs from the control group (**a**) and treatment group (**b**), and F-actin with FITC-labeled phalloidin in BMSCs from the control group (**c**) and treatment group (**d**) at 24 h after mitochondria transfer. Confocal immunofluorescence staining of transferred mitochondria labeled before isolation from the control group (**e**) and treatment group (**f**) at 24 h after mitochondria transfer. **g**, **h** Merged confocal images of **a**, **c**, and **e** or **b**, **d**, and **f**, respectively. Scale bar represents 50 μm. Confocal immunofluorescence imaging of treatment groups containing amplified xZ-stack and yZ-stack images (**i**). **j** FACS analysis of the number of labeled mitochondria (displayed as fluorescence intensity per cell) within recipient cells of each group. **k** Linear regression showing linear relationship between relative MFI values and labeled mitochondria number. Results are presented as mean ± SEM (*n* = 3)

In order to validate whether isolated mitochondria can be effectively transferred into BMSCs, we labeled mitochondria in donor BMSCs (donor-cell/recipient-cell ratio: 1:1) with MitoTracker dye before isolation. Twenty-four hours after mitochondria transfer, fluorescence from the donor mitochondria was not detected in the control cells (Fig. 1e), but was observed in the recipient cells (Fig. 1f, i). This thus indicates that mitochondria could be successfully transferred into BMSCs. To determine if there was any dose-dependency, mitochondria were isolated from 1/8, 1/4, 1/2, the same amount, or 2 times the number of cells for each group (donor-cell/recipient-cell ratios: 0.125, 0.25, 0.5, 1, 2), and transferred into recipient cells. Cells in the control group (Con) were treated with culture medium without any isolated mitochondria instead. Mitochondria were also labeled with MitoTracker® before isolation and the mean fluorescence intensity (MFI) of recipient cells or control cells was quantified by FACS. The percentage of positive cells in the control group was set to lower than 0.10%, and the percentage of positive cells showed a dose-dependent tendency, with a gradual increase from 4.43 to 90.90% in the five groups (Fig. 1j). A linear relationship between relative MFI values and labeled mitochondria number further validated the above results (Fig. 1k). Hence, it was clearly demonstrated that mitochondria could be artificially transferred into BMSCs in vitro and that within certain limits, the more mitochondria that were transferred, the more the recipient cells were able to receive.

### Mitochondria transfer enhanced the proliferative capacity of BMSCs in vitro

**Fig. 2:**
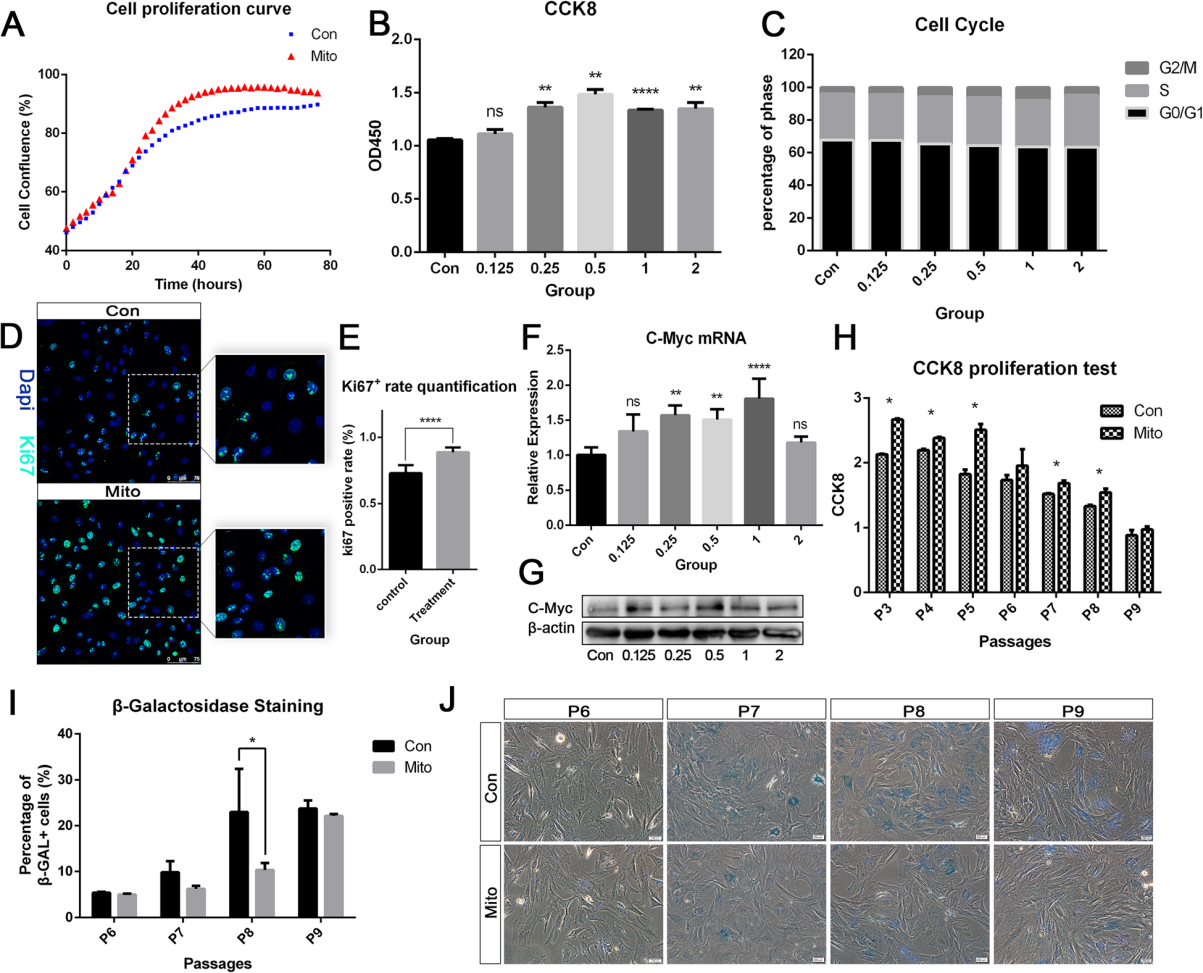
Mitochondria transfer enhanced the proliferative capacity, as demonstrated by **a** real-time plot of cell confluency, **b** CCK8 assay results, and **c** cell cycle analysis results, presented as percentages of cells in G2/M, S, or G0/G1 phase. **d** Differences in Ki67 staining of BMSCs between the control and mitochondria transfer group, and **e** quantification of the percentage of Ki67-positive cells within the entire cell population. **f** mRNA expression level of proliferation-related marker c-Myc and **g** its corresponding protein expression level. **h**–**j** Effects of mitochondria transfer on replicative senescence, as shown by **h** CCK8 assay results at the 3rd to 9th passages, **j** β-galactosidase staining, and **i** percentages of β-GAL-positive cells at the 6th to 9th passages. Results are presented as the mean ± SEM (*n* = 3). One-way ANOVA with Tukey’s post hoc test was used to determine statistical significance (*P ≤ 0.05, **P ≤ 0.01, ****P ≤ 0.0001)

BMSCs require strong proliferative capacity in order to be amplified into suitable numbers for transplantation therapy. We investigated how mitochondria transfer influence the proliferative capacity of BMSC in vitro. Firstly, we observed the real-time change of cell confluency after mitochondria transfer, starting from the same cell confluency of around 45%. The treatment group reached plateau earlier than the control group and showed higher cell confluency at the same observation timepoint after 20 h (Fig. 2a). The CCK8 assay was performed 24 h after mitochondria transfer, whereby BMSCs in the various treatment groups (0.25, 0.5, 1, and 2) all showed significantly higher proliferative potential (*P* < 0.05, *n* = 3), with the best result in group 1 (*P* < 0.001, *n* = 3; Fig. 2b). We then investigated whether there exist certain changes in the cell cycle, and positive changes were confirmed by cell cycle analysis. Cells receiving mitochondria tended to transit more into the S and G2/M phase rather than the G1/G0 phase, as compared to control cells, cells in group 1 exhibiting the longest G2/M phase compared to other groups, even though differences were non-significant (Fig. 2c). Moreover, Ki67 staining showed that more actively proliferative cells were observed after mitochondria transfer (Fig. 2d, e). C-Myc is an oncogene involved in orchestrating changes in cell metabolism necessary for cell-cycle entry in mitotic cells [18]. Since c-Myc expression has been proven to promote proliferation rates of MSC in previous studies [19, 20], we evaluated the mRNA and protein expression levels of c-Myc. Notably, c-Myc mRNA expression was significantly upregulated in all groups, reaching a peak in group 1 (Fig. 2f). Similarly, the expression of c-Myc protein was also upregulated in all mitochondria transfer groups (Fig. 2g). Since MSCs might enter senescence after long-term or continuously passage cultivation in vitro [21], we investigated whether mitochondria transfer could possibly rescue BMSCs from replicative senescence. After mitochondria transfer, BMSCs were cultivated from passage 3 to 9, mitochondria-recipient BMSCs at passage 3–5 and 7–8 displayed significantly higher CCK8 result than the control group (Fig. 2h). β-Galactosidase (β-GAL) staining was used to detect senescent cells. The percentages of β-GAL-positive cells in mitochondria-recipient BMSCs were lower at passage 7–9, with significant difference at passage 8 (Fig. 2i, j). The two results above indicated that the upregulation effect of mitochondria transfer on proliferation lasted for at least 5 passages. Therefore, it can be concluded that mitochondria transfer effectively enhanced the proliferative capacity and resisted the replicative senescence of BMSCs.

### Migration of BMSCs was enhanced by mitochondria transfer

**Fig. 3:**
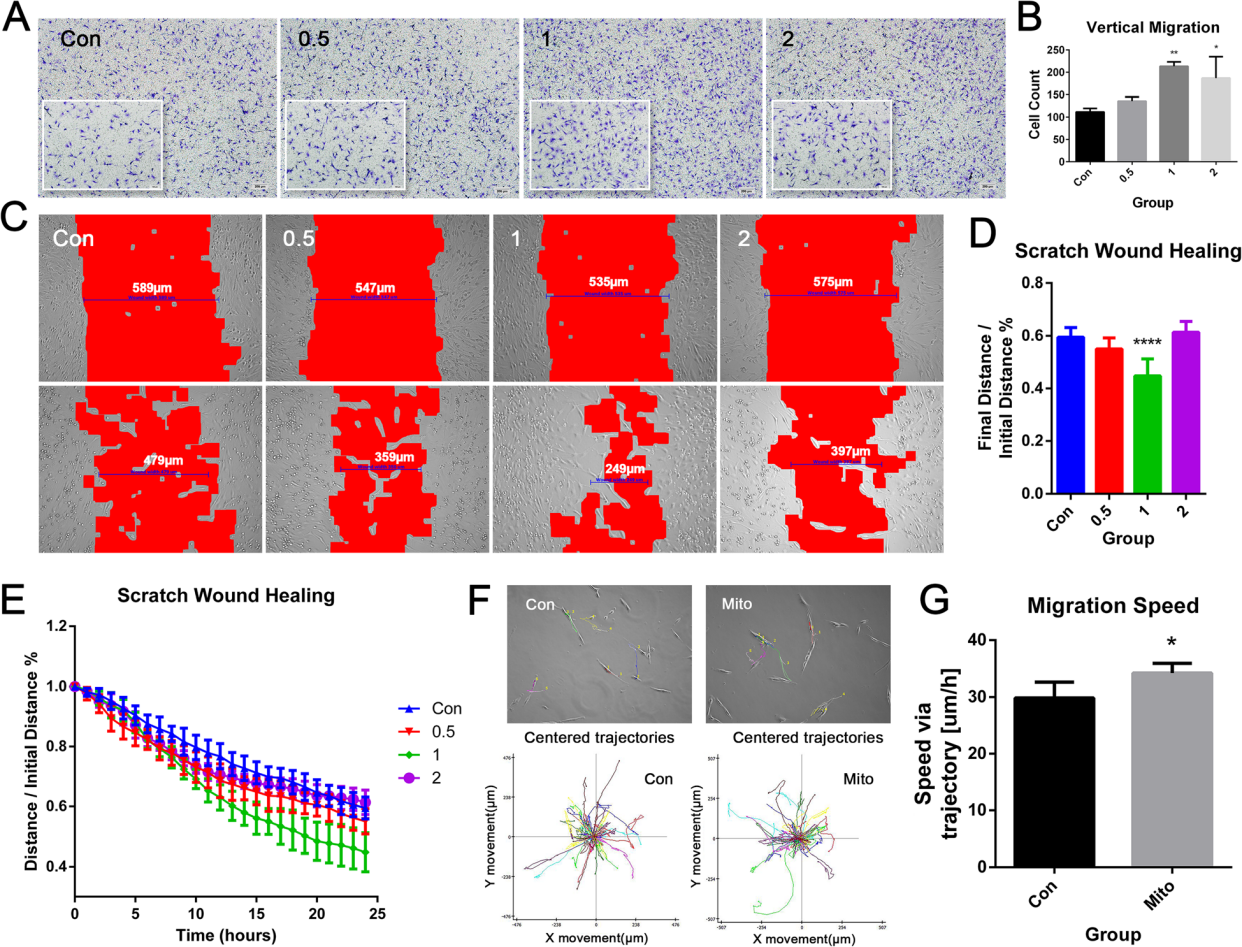
Mitochondria transfer improved the migration and invasive capacities of BMSCs. **a**, **b** Effects of mitochondria transfer on vertical migration, as shown by × 4 and × 10 (in the lower left corner) micrographs of BMSCs in different groups at the bottom of the migration trans-well culture chamber (**a**), and histogram of cell counts based on × 10 magnification field (**b**). Results are presented as the mean ± SEM (*n* = 3). **c**–**e** Effects of mitochondria transfer on lateral migration, as shown by the initial and final micrographs of the scratch in the wound healing assay (**c**), bar graph of final distance/initial distance rate of each group (**d**), and line chart of real-time scratch distance (**e**). Results are presented as the mean ± SEM (*n* = 6). **f**, **g** Single cell tracking of control or mitochondria-recipient BMSCs, showing cell migration trajectory in the colored lines (**f**), and bar graph of quantitative migration speed (**g**). Results are presented as the mean ± SEM (*n* = 6). One-way ANOVA with Tukey’s post hoc test was used to assess statistical significance (*P < 0.05, **P < 0.01, ***P < 0.001)

The transwell migration assay and scratch wound healing test were performed to examine the vertical migration and lateral migration capacities of BMSCs, respectively, in vitro. Cells receiving mitochondria transfer were observed to have stronger vertical (Fig. 3a, b) and lateral migration capacities (Fig. 3c–e), as compared to control BMSCs. Quantification showed that group 1 had the highest migration capacity, with significant differences compared to the control group (Fig. 3b, d). Measurements of real-time scratch distance showed that mitochondria transfer accelerated the wound healing process, particularly in group 1 (Fig. 3e). As shown in Fig. 3f, cell migration pathways were labeled with lines of different colors, with each line representing the migration trajectory of one single cell over a 12-h period. Cell migration speed (trajectory divided by time) in the mitochondria transfer group was significantly higher than that of the control group (*n* = 6, Fig. 3g). Hence, mitochondria transfer significantly enhanced the migration of BMSCs, possibly indicating its positive effects on tissue repair by improving stem cell homing.

### Mitochondria transfer improved osteogenic potential of BMSCs in vitro

**Fig. 4:**
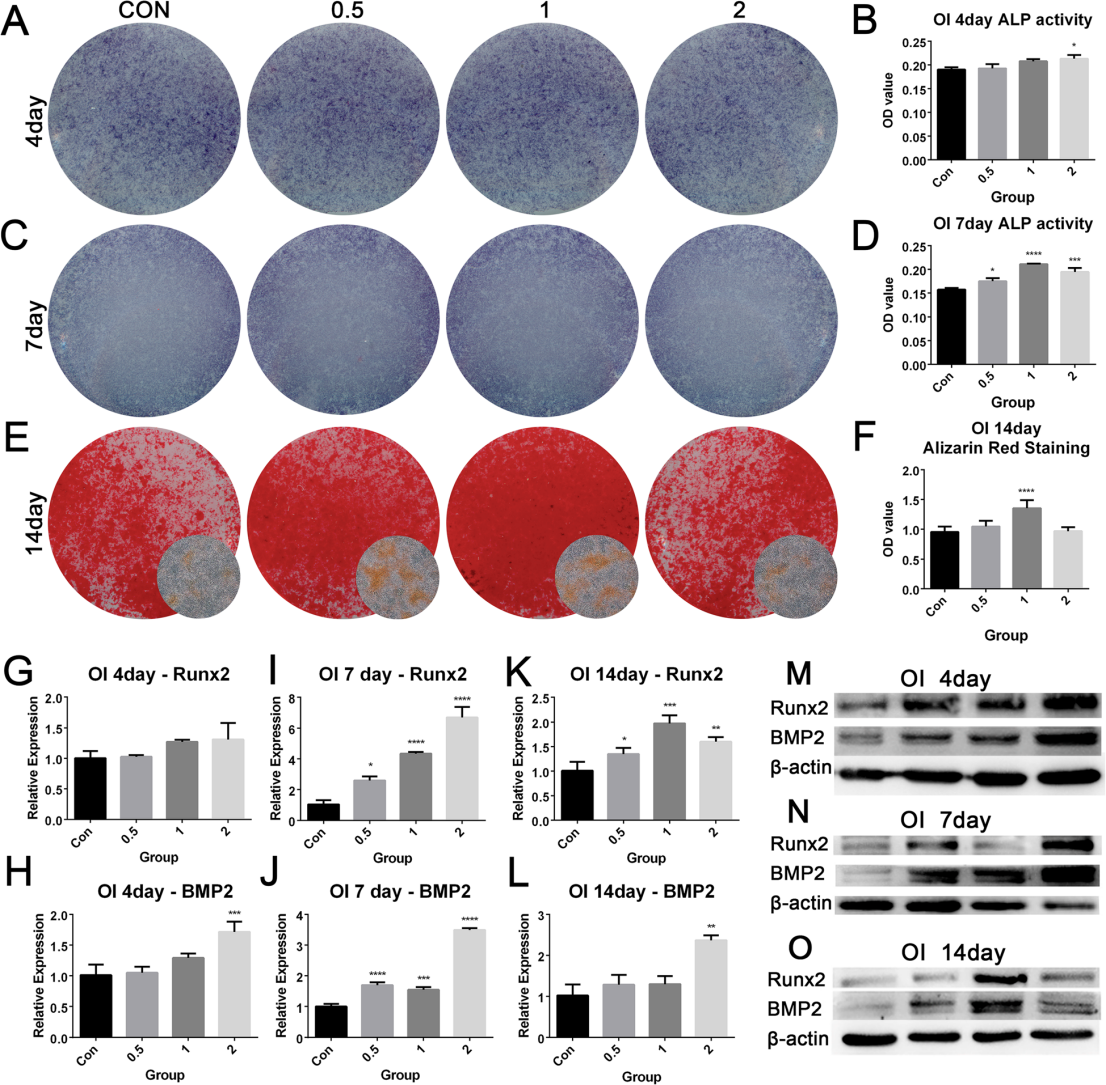
Mitochondria transfer enhanced the osteogenic differentiation of BMSCs in vitro. Gross images of alkaline phosphatase staining (**a**, **c**) and ALP activity levels (**b**, **d**) of mitochondria-recipient or control BMSCs at 4 days (**a**, **b**) or 7 days (**c**, **d**) after osteogenic induction (OI). Gross images and micrographs (**e**) of Alizarin Red staining of mitochondria-recipient or control BMSCs at 14 days after OI and their quantitative measurement of mineralization (**f**). The mRNA expression levels of Runx2 (**g**) and BMP2 (**h**) and their corresponding protein expression levels (**m**) in BMSCs of the different groups after 4 days of OI. The mRNA expression levels of Runx2 (**i**) and BMP2 (**j**) and their corresponding protein expression levels (**n**) in BMSCs of the different groups after 7 days of OI. The mRNA expression levels of Runx2 (**k**) and BMP2 (**l**) and their corresponding protein expression levels (**o**) in BMSCs of the different groups after 14 days of OI. Results are presented as the mean ± SEM (*n* = 3). One-way ANOVA with Tukey’s post hoc test was used to determine statistical significance (*P < 0.05, **P < 0.01, ***P < 0.001, ****P ≤ 0.0001)

BMSCs can be induced to differentiate into osteoblasts in vitro, with a chemical cocktail of dexamethasone, ascorbate, and β-glycerophosphate [8]. Activation of Runx2 nuclear transcription factor and bone morphogenetic proteins (BMPs) are related to the osteogenic pathway of MSCs [8]. Hence, we subjected BMSCs to osteogenic induction after mitochondria transfer and observed significantly increased osteogenesis effects in group 1 on the 7th and 14th day of induction, as demonstrated by increased ALP staining, ALP activity assay, and Alizarin Red staining (Fig. 4c–f), which was verified by qPCR analysis of the mRNA expression levels of Runx2 (Fig. 4i, k), as well as Western blot protein expression levels of Runx2 and BMP2 (Fig. 4m–o). Runx2 and BMP2 expression levels also displayed an upward trend from the control group to 0.5, 1, and 2 treatment groups on the 4th day of induction (Fig. 4g, h), with or without significant differences. Overall, the osteogenic potential of BMSCs was improved by mitochondria transfer.

### Effects of mitochondria transfer on bone defect repair and healing in vivo

**Fig. 5:**
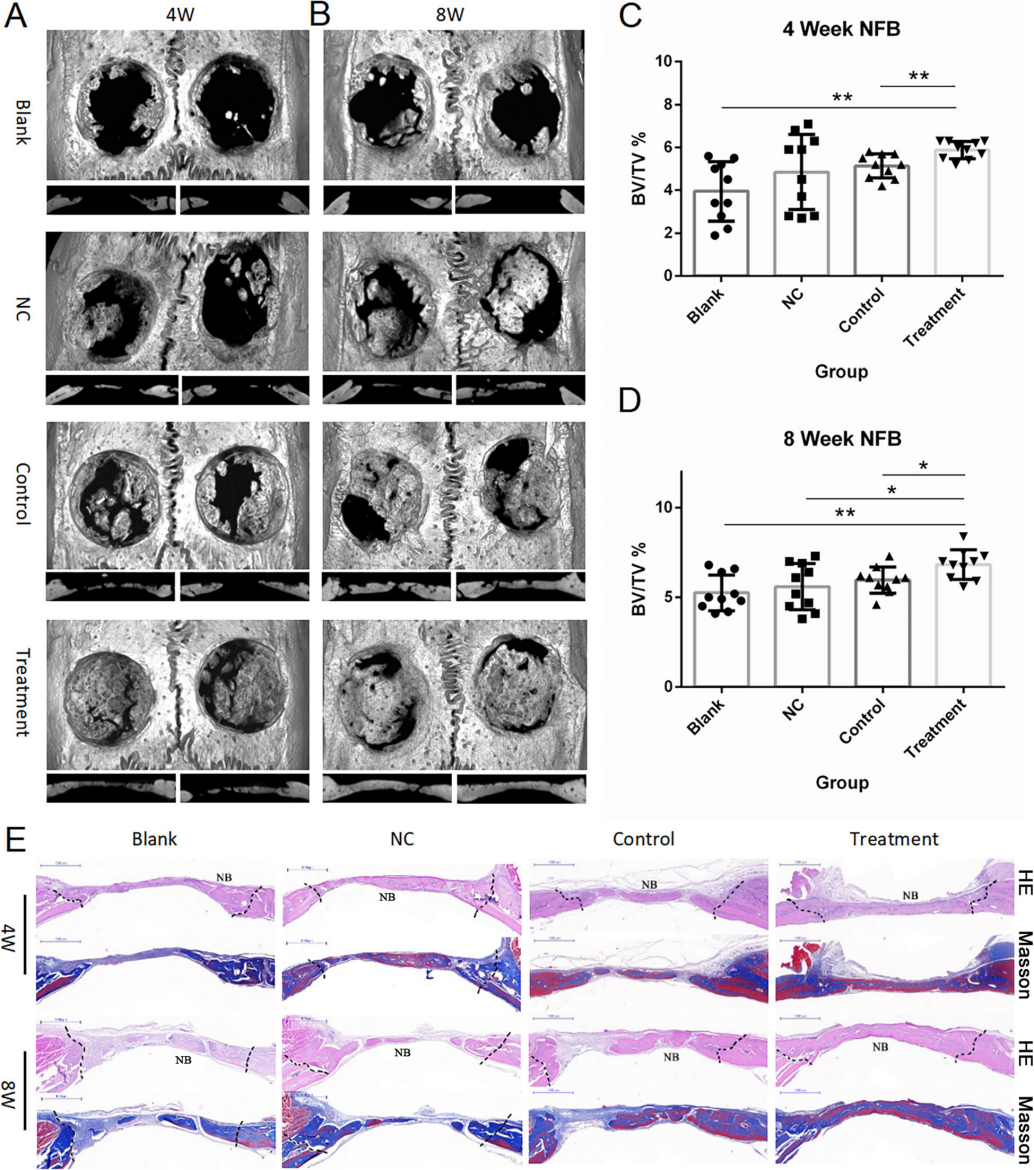
Mitochondria transfer enhances the therapeutic effects of BMSCs on bone defect healing in vivo. **a**, **b** Representative micro CT images and sagittal views of rat cranial critical-sized full-thickness defects at 4 (**a**) and 8 weeks (**b**) after surgery. **c**, **d** Quantitative analysis of bone volume (BV) and bone mineral density (BMD) of the newly formed bone. One-way ANOVA with Tukey’s post hoc test was used to assess statistical significance (*P < 0.05, **P < 0.01, ***P < 0.001). **e** Scans of HE or Masson staining of bone tissue slices of specimens (NB, nascent bone)

Given that osteogenic differentiation potential was increased by mitochondria transfer in vitro, it is reasonable to hypothesize that mitochondrial transfer might also influence how BMSCs facilitate the repair of critical-sized bone defects in vivo. Hence, we transplanted control BMSCs or mitochondria-recipient BMSCs into rat cranial bone defects and observe the bone healing process visually and histologically. Micro CT scanning of rat skulls showed that more new bone was formed within the 5-mm defect area in the treatment group, as compared to the blank, NC, or control groups, at both 4 weeks and 8 weeks post-surgery (Fig. 5a, b). Quantitative statistics were carried out to calculate the NFB (newly formed bone) of each group and significant differences were observed in the treatment versus control groups at both 4 and 8 weeks (Fig. 5c, d). Through the tissue slicing and Masson staining techniques, we observed a larger area of newly formed bone, which contained more collagen tissue (Fig. 5e) in the treatment versus control groups. It can therefore be concluded that mitochondria transfer does enhance bone defect repair in vivo.

### Mitochondria transfer enhanced functions of BMSCs through increased OXPHOS activity and ATP production of recipient cells

**Fig. 6:**
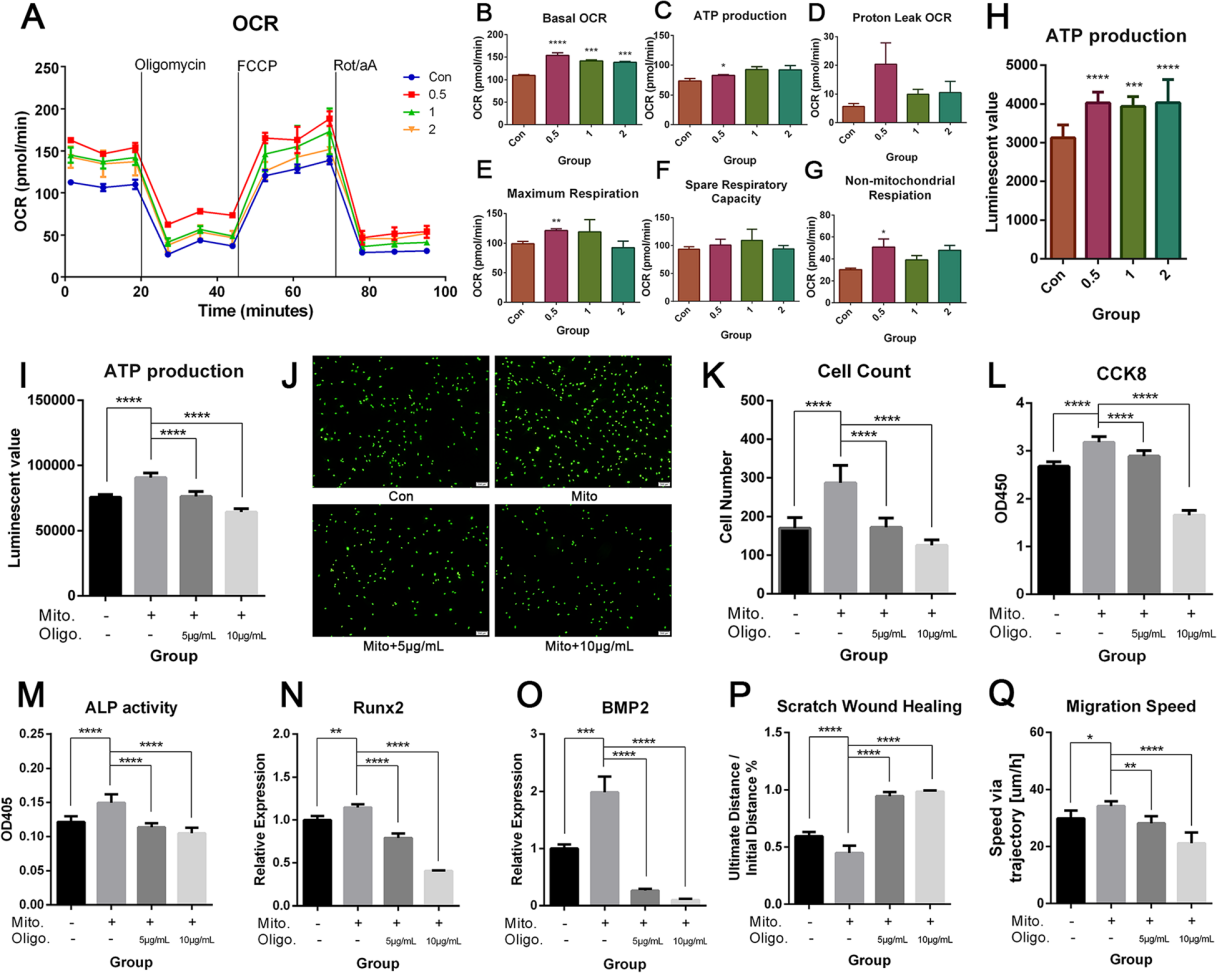
Effects of mitochondria transfer on BMSC metabolism. The extracellular flux analysis of OXPHOS activity in mitochondria-recipient BMSCs versus control, including **a** measurements of oxygen consumption rates (OCR, pmols O2/min) throughout the entire detection process, and **b**–**g** calculations of mean OCR at different stages correlated to basal OCR (**b**), ATP production (**c**), proton leak OCR (**d**), maximum respiration (**e**), spare respiratory capacity (**f**), and non-mitochondrial respiration (**g**). Measurement of ATP production (**h**) by ATP luminescent detection assay. **i** Effects of ATPase inhibitor oligomycin on ATP production of recipient BMSCs. Representative micrographs of fluorescently labeled cells (**j**), quantitative results of fluorescent cell count (**k**), and changes in CCK8 absorbance readings (**l**) after treating mitochondria-recipient BMSCs with 5 or 10 μg/mL of oligomycin for 12 h. Effects of 5 or 10 μg/mL oligomycin treatment on the osteogenic differentiation potential of mitochondria-recipient BMSCs, as assessed by ALP activity (**m**), mRNA expression levels of Runx2 (**n**), and BMP2 (**o**) after 4 days of osteogenic induction. Effects of 5 or 10 μg/mL oligomycin treatment on the migration capacity of recipient BMSCs, as evaluated by the scratch wound healing assay (**p**) and the Cell-IQ single cell migration speed test (**q**). Results are presented as the mean ± SEM (*n* = 3). One-way ANOVA with Tukey’s post hoc test was used to assess statistical significance (*P < 0.05, **P < 0.01, ***P < 0.001, ****P < 0.0001)

In order to uncover the underlying mechanisms of the observed enhancements in proliferation, migration capacities, and osteogenic differentiation after mitochondria transfer, cellular OXPHOS activity after mitochondria transfer was investigated next. The whole cell OXPHOS level increased after mitochondria transfer (Fig. 6a), with group 0.5 exhibiting the highest general oxygen consumption rate (OCR) throughout the process. As shown in Fig. 6b, basal OCR was significantly increased in the mitochondria transfer groups, as compared to the control, thus indicating an increase in the respiration capacity at rest. The ATP production-related OCR was significantly higher in group 1 (Fig. 6c), and the ATP production capacity measured by the SeaHorse Flux Analyzer correlated with the results of the ATP detection assay, which showed massive augmentation of ATP production in the mitochondria transfer groups (Fig. 6h). The maximum respiration potential and non-mitochondrial respiration capacity were also increased in group 0.5 (with significant difference) and group 1 (Fig. 6e, g). Finally, the proton leak OCR and spare respiratory capacity exhibited a modest increase in the mitochondria transfer groups, even though differences were not significantly different (Fig. 6d, f). The relationship between ATP production and functional changes was evaluated through oligomycin (ATPase inhibitor) treatment. After treating mitochondria-recipient BMSCs with 5 μg/mL or 10 μg/mL oligomycin, ATP production was significantly decreased (Fig. 6i). The increase in cellular proliferation following mitochondria transfer, as measured by the CCK8 test, was counteracted by 5 μg/mL oligomycin and even downregulated by 10 μg/mL oligomycin, as compared to the control (Fig. 6j). This was consistent with results of the CyQUANT™ cell proliferation assay, which stained the DNA for cell quantification. The cell number was increased after mitochondria transfer, but was reduced to the same level as control BMSCs by 5 μg/mL oligomycin treatment, and further lowered by 10 μg/mL oligomycin (Fig. 6k, l). Lower ALP activity (Fig. 6m) and lower Runx2 and BMP2 mRNA expression levels (Fig. 6n, o) were observed in BMSCs treated with either 5 or 10 μg/mL oligomycin after mitochondria transfer, thus demonstrating decreased osteogenic potential caused by lower OXPHOS activity and ATP production. Reduced migration capacity induced by oligomycin treatment was subsequently observed in the scratch wound healing experiment and single cell motility test, thus indicating that enhanced migration capacity after mitochondria transfer was impaired by oligomycin (Fig. 6p, q). Hence, we came to the conclusion that mitochondria transfer enhanced proliferation, osteogenic differentiation potential, and migration capacity by increasing cellular aerobic metabolic levels and mitochondrial ATP production.

## Discussion

Enhancing BMSCs functions is a critical step in optimizing stem cell-mediated bone repair. Firstly, increased proliferative capacity enables MSCs to be expanded in vitro to sufficient numbers for clinical transplantation [22]. Secondly, after being engrafted, it is of utmost importance for BMSCs to continuously proliferate and migrate to injury sites [23, 24] and either differentiate into osteoblasts [25] or secrete trophic factors to stimulate targeted cells [26, 27]. However, various factors such as aging and pathological conditions might affect MSCs survival or functions after transplantation and therefore reduce their therapeutic effects [6, 28]. Hence, we investigated mitochondrial transfer as a novel strategy to overcome these limitations.

To our knowledge, this study is the first to transfer autologous mitochondria into BMSCs and evaluate its effects on functional cellular changes. Our results conclusively demonstrated that mitochondria transfer could significantly increase proliferation, osteogenesis, and migration of BMSCs in vitro. OXPHOS activity and mitochondrial ATP production were found to be upregulated after mitochondria transfer. Furthermore, we transplanted the mitochondria-recipient BMSCs into rat cranial bone defect sites and found that mitochondria transfer could accelerate the bone defect healing process mediated by BMSCs.

The safety issues of genetic modification techniques have always been an intractable challenge in stem cell-based tissue engineering [5, 14, 29–31]. For example, the viral vector used in gene therapy has genotoxicity issues [32], and the off-target mutations or effects of techniques like CRISPR-Cas9 (clustered regularly interspaced short palindromic repeats, associated RNA guided endonuclease Cas9, [33, 34]) or RNA interference [35] might exert detrimental toxic effects and induce unwanted phenotypes. Alternatively, the functions of BMSCs may be enhanced by treatment with growth factors or small molecule drugs, but these also have intrinsic drawbacks such as unclear safe dosage range, possible side effects, or ectopic influences [6]. For instance, even BMP-2 (bone morphogenetic protein-2), the only current FDA (Food and Drug Administration)-approved osteo-inductive growth factor, has been reported to exert numerous side effects that can result in potentially devastating complications such as ectopic bone formation, osteoclast-mediated bone resorption, and inappropriate adipogenesis, which tend to manifest at higher concentration [36, 37]. Hence, the various aforementioned drawbacks impede the clinical translation of these potentially-useful therapeutic tools.

However, mitochondria transfer can circumvent biosafety concerns due to the following reasons. First and foremost, mitochondria are intrinsic cellular organelles that are ubiquitously present in all eukaryocytes [38]. In our study, we transferred autologous mitochondria isolated from the same batch of cells, since all available scientific data have shown that autologous mitochondria transplantation does not provoke any auto-immune responses, thus indicating that it is immunologically safe [39]. Moreover, the transfer of mitochondria is believed not to involve any transfer of nuclear materials, which would thus allay safety concerns relating to nuclear genomic modification [40]. More importantly, mitochondria transfer can modulate BMSCs function without any changes to the extracellular microenvironment, unlike treatment with drugs, growth factors, or biomaterials, thus avoiding any possible safety concerns pertaining to cytotoxicity or biocompatibility. Therefore, mitochondria transfer should be considered a rather safe technique for modulating BMSCs function. In our study, mitochondria isolated from the same batch of cells was demonstrated to exert the strongest effects. The procedure of isolating and transferring mitochondria has been proven to be simple and not too time-consuming, with relatively high success rates. As compared to other enhancement strategies, mitochondria transfer is more easily controllable, stable, and effective.

It has been observed in our study, as well as other studies, that the acquisition of additional mitochondria during transfer results in an increase in OXPHOS activity and ATP production of mitochondria-recipient MSCs [41, 42]. The increased aerobic metabolic levels of BMSCs might then contribute to the enhancement of proliferation, osteogenic, and migratory functions. There are several possible explanations for these observed changes in cellular function. During the process of proliferation and colony formation in vitro, which usually occurs under normoxic conditions (around 20% O_2_ tension), MSCs rely more on OXPHOS for energy supply rather than glycolysis [43], and the proliferative process of cells, particularly cell-cycle entry, requires increased oxygen consumption and ATP generation [44]. Cell differentiation is also associated with an increase in mitochondrial content and activity, according to previous studies [45–47]. The activation of mitochondrial OXPHOS in BMSCs is known to trigger osteogenic differentiation via acetylation and activation of β-catenin signaling [48]. The relationship between BMSC migration and cellular energy metabolism has yet to be investigated. However, cancer cells were found to be expending energy via the dephosphorylation of ATP into ADP during the metastatic process [49]. In migrating ovarian cancer cells, mitochondria actively infiltrate the leading edge of the lamellipodia, increasing the local mitochondrial mass and relative ATP concentration [50]. Thus, it can be hypothesized that mitochondria transfer enhanced BMSC functions through the upregulation of aerobic respiratory levels. In order to validate our hypothesis, we utilized oligomycin, an ATP synthase (mitochondria respiratory chain complex V) inhibitor, to attenuate OXPHOS and ATP production in BMSCs, and found that any enhancement of proliferation, differentiation, and migration by mitochondria transfer was eliminated. This finding thus proved that mitochondria transfer enhanced BMSCs proliferation, osteogenic differentiation, and migration through upregulation of OXPHOS activity and ATP production.

Nevertheless, there are still a number of limitations to our study. Firstly, although transplantation of mitochondria-recipient BMSCs resulted in stronger bone regeneration efficacy compared to transplantation of control BMSCs, the underlying mechanisms still remain unclear. Because an increasing number of reports emphasized the paracrine effects of MSCs on tissue regeneration, further investigations of the crosstalk between mitochondria-recipient BMSCs and other cell types (e.g., macrophages or endothelial cells) after transplantation need be performed. Secondly, although our data demonstrated the key role of increased aerobic metabolism in regulating BMSCs function after mitochondria transfer, other mechanisms that elicit functional modification of BMSCs also need to be further investigated. Thirdly, there is an obvious limit to the number of BMSCs that can be isolated from each individual patient, which could in turn impede the clinical application of autogenous mitochondria transfer between BMSCs from the same patient. Hence, our future studies would investigate mitochondria transfer between different patient and tissue sources. For example, autogenic mitochondria transfer between adipose MSCs and BMSCs from the same patient, or even allogeneic mitochondria transfer from the BMSCs of younger patients to that of older patients.

## Conclusions

In conclusion, we have provided firm evidences that mitochondria transfer can be a feasible technique to enhance the proliferative capacity, osteogenic potential, and migration capacity of BMSCs in vitro through the upregulation of aerobic metabolism, as well as further demonstrated that mitochondria transfer promoted bone defect repair in situ. These findings might thus provide a novel strategy to improve BMSC function, prior to being utilized in transplantation and tissue engineering.

## Data Availability

All data generated or analyzed during this study are included in this published article.

## Abbreviations

BMSCs: Bone marrow mesenchymal stem cells
CCK8: Cell Counting Kit-8
ALP: Alkaline phosphatase
Runx2: Runt-related transcription factor 2
BMP2: Bone morphogenetic proteins-2
OXPHOS: Oxidative phosphorylation
ATP: Adenosine Triphosphate
MSC: Mesenchymal stem cells
TGF-β1: Transforming growth factor-β1
TNF-α: Tumor necrosis factor-α
CXCR4: Chemokine receptor 4
MFI: Mean fluorescence intensity
β-GAL: β-Galactosidase
OCR: Oxygen consumption rate
